# The Efficacy of Magnetic Resonance Imaging and Color Doppler Ultrasonography in Diagnosis of Salivary Gland Tumors

**DOI:** 10.5681/joddd.2014.044

**Published:** 2014-12-03

**Authors:** Behrooz Davachi, Mahrokh Imanimoghaddam, Mohamad Reza Majidi, Ahmad Sahebalam, Masoomeh Johari, Adineh Javadian Langaroodi, Mohamad Taghi Shakeri

**Affiliations:** ^1^Assistant Professor, Department of Radiology, Ghaem Hospital, Mashhad University of Medical Sciences, Mashhad, Iran; ^2^Professor, Oral and Maxillofacial Diseases Research Center, Mashhad University of Medical Sciences, Mashhad, Iran; ^3^Assistant Professor, Department of Otorhinolaryngology, Ghaem Hospital, Mashhad University of Medical Sciences, Mashhad, Iran; ^4^Associate Professor, Department of Radiology, Ghaem Hospital, Mashhad University of Medical Sciences, Mashhad, Iran; ^5^Assistant Professor, Department of Oral and Maxillofacial Radiology, Faculty of Dentistry, Tabriz University of Medical Sciences, Tabriz, Iran; ^6^Assistant Professor, Department of Oral and Maxillofacial Radiology, Faculty of Dentistry, Mashhad University of Medical Sciences, Mashhad, Iran; ^7^Professor, Department of Biostatistics, Mashhad University of Medical Sciences, Mashhad, Iran

**Keywords:** Color, doppler, magnetic resonance imaging, salivary gland neoplasms, ultrasonography

## Abstract

***Background and aims.*** Although salivary gland tumors are not very common, early diagnosis and treatment is crucial because of their proximity to vital organs, and therefore, determining the efficacy of new imaging procedures becomes important. This study aimed to evaluate the efficacy of magnetic resonance imaging (MRI) and color doppler ultrasonography parameters in the diagnosis and differentiation of benign and malignant salivary gland tumors.

***Materials and methods.*** In this cross-sectional study, color doppler ultrasonography and MRI were performed for 22 patients with salivary gland tumor. Demographic data as well as MRI, color doppler ultrasonography, and surgical parameters including tumor site, signal in MRI images, ultrasound echo, tumor border, lymphadenopathy, invasion, perfusion, vascular resistance index (RI), vascular pulse index (PI) were analyzed using Chi-square test, Fisher's exact test, and independent t-test.

***Results.*** The mean age of patients was 46.59±13.97 years (8 males and 14females). Patients with malignant tumors were older (P < 0.01). The most common tumors were pleomorphic adenoma (36.4%), metastasis (36.4%), and mucoepidermoid carcinoma (9%). Nine tumors (40.9%) were benign and 13 (59.1%) were malignant. The overall accuracy of MRI and color doppler ultrasonography in determining tumor site was 100% and 95%, respectively. No significant difference observed between RI and PI and the diagnosis of tumor.

***Conclusion.*** Both MRI and ultrasonography have high accuracy in the localization of tumors. Well-identified border was a sign of benign tumors. Also, invasion to adjacent structures was a predictive factor for malignancy.

## Introduction

Salivary gland tumors are not very common and only account for less than 3% of all head and neck neoplasms. The majority of salivary gland tumors are low grade or benign and in most cases symptomless. Approximately 70-80% of salivary gland tumors occur in parotid gland where 17-34% have shown malignancy.^1,2^

Clinical signs and symptoms predicting benign or malignant nature of tumors are nonspecific. However, most benign or low grade malignant tumors are presented as painless masses with slow growth. Progressive malignancies or inflammations are accompanied with pain and rapid growth. Facial nerve paralysis associated with parotid mass is highly predictive of malignancy and poor prognosis. However, benign mixed tumors accompanied with facial nerve paralysis are also reported.^1,2^

There have been controversial data regarding the role of imaging prior to surgery for parotid tumors. Although some studies recommend fine needle aspiration (FNA) alone for the evaluation of superficial parotid masses prior to superficial parotidectomy, others suggest imaging procedures before surgery.^3,4^ It is important to determine whether the tumor is intra or extra glandular as well as superficial or deep in advance of the surgical procedures; therefore, using imaging techniques help not only in planning the operation but also in evaluating further possible relapses and complications.^2^ Although various imaging techniques have been used to evaluate salivary gland tumors, ultrasonography has received significant attention in cases where it could only reveal superficial tumors while the relation of tumor to other adjacent structures could not be identified. However, it is still a cost effective non-invasive technique.^1,2,5^

Color doppler ultrasonography was recently introduced as an imaging technique for the evaluation of salivary gland tumors as well as vascular system and it is believed to have future applications in this field.^1^ A study on capabilities of ultrasound in salivary gland tumors showed B-mode ultrasonography and color doppler imaging could reveal the presence of masses in the gland, their topography and dimensions, and specific vascularization, which are effective in treatment planning.^5^ Zengel et al^6^ stated ultrasound examination alone is sufficient to diagnose benign tumors, however, other techniques such as computed tomography or magnetic resonance imaging (MRI) may also be required in malignant tumors to determine infiltration to the skull base.^6^

MRI is recommended by many studies as an accurate imaging technique to evaluate tumors and adjacent soft tissue structures and also to study the associations between them.^1,7^ Also the results of a study revealed that gadolinium-enhanced dynamic MRI could help in differentiating benign from malignant parotid gland tumors and characterizing the pathologic types of benign tumors.^8^ This study was aimed to evaluate the efficacy of MRI and color doppler ultrasonography parameters in the diagnosis and differentiation of benign and malignant salivary gland tumors.

## Materials and Methods

In this cross-sectional study, 22 patients who were suspected to have salivary gland tumor and were referred to one of the three referral centers including the Department of Oral Medicine at the Faculty of Dentistry, Ghaem Hospital, and Omid Hospital, all located in Mashhad, Northeast of Iran, during an 18-month period were enrolled. MRIs and color coppler ultrasonographies were performed for 22 and 20 patients, respectively. The site of tumor located in the palate made it impossible for the radiologist to perform ultrasonic evaluations in two patients.

This study was approved by the research deputy of Mashhad University of Medical Sciences regarding methodological and ethical issues. A written consent was obtained from each individual prior to the procedures. The aims of the study were explained to the participants and their questions were answered.

Philips MRI system (Philips, The Netherlands) with 0.5 Tesla power was used to produce T1, T2, coronal, and axial images with 1-4 mm section thickness. All ultrasonographies performed using Toshiba Ultrasound system (Toshiba, Japan) with 7.5 MHz power.

MRI images were reviewed by two radiologists who were not informed of the histopathological diagnoses. Evaluated parameters in MRI were tumor site, signals of T1 and T2 images, tumor border, tumor homogenicity, lymphadenopathy, and invasion to adjacent structures. Tumors sites were classified into parotid (the ramus of the mandible was used as an index to differentiate superficial and deep tumors as if the tumor grows to the medial portion of ramus it would be considered as deep), submandibular, sublingual, and minor salivary glands.

**Figure 1.**
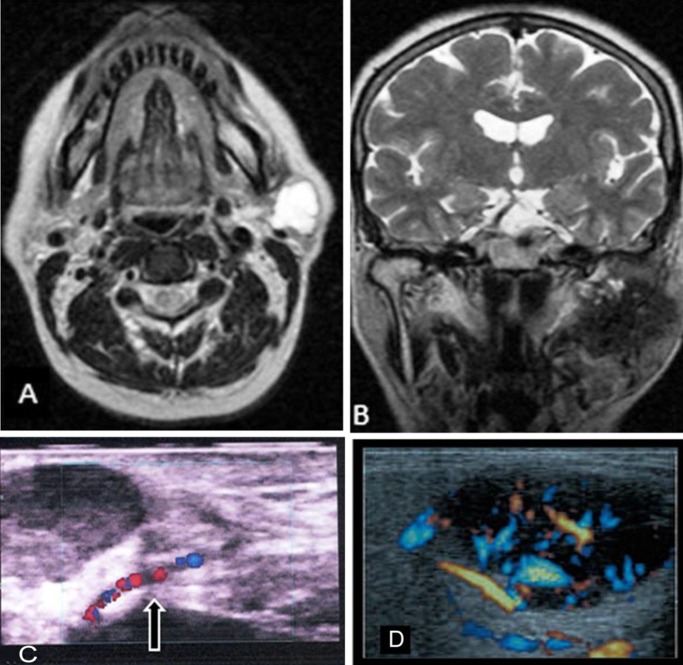
(A) Axial view of a high signal pleomorphic adenoma in left parotid; (B) Coronal view of a low signal malignant oncocytoma in left parotid; (C) Pleomorphic adenoma with no perfusion. Note the signal flow related to the facial artery (arrow). (D) Warthin tumor with +++ perfusion scale and mixed (central and peripheral) perfusion pattern.

Tumor border was classified as well-defined, and ill-defined. T1 and T2 signals were categorized into hypointense if tumor signals were lower than adjacent muscles, isointense if signals were analogous, and hyperintense if tumor signals were higher than adjacent muscles (Figure 1). Homogenicity was classified as homogenous and heterogenous.

Also all documents of invasion to adjacent structures as well as metastases were registered.

Ultrasound data included tumor site, echo, border, vascular resistance index (RI), vascular pulse index (PI), perfusion pattern and scale, and lymphadenopathy.

Tumor echo was classified as hypoechoic, hyperechoic, and complex echo according to the comparison with normal tissue echo pattern. Complex echo was defined as a combination of hyperechoic and hypoechoic patterns in the tumor site.

**Figure 2.**
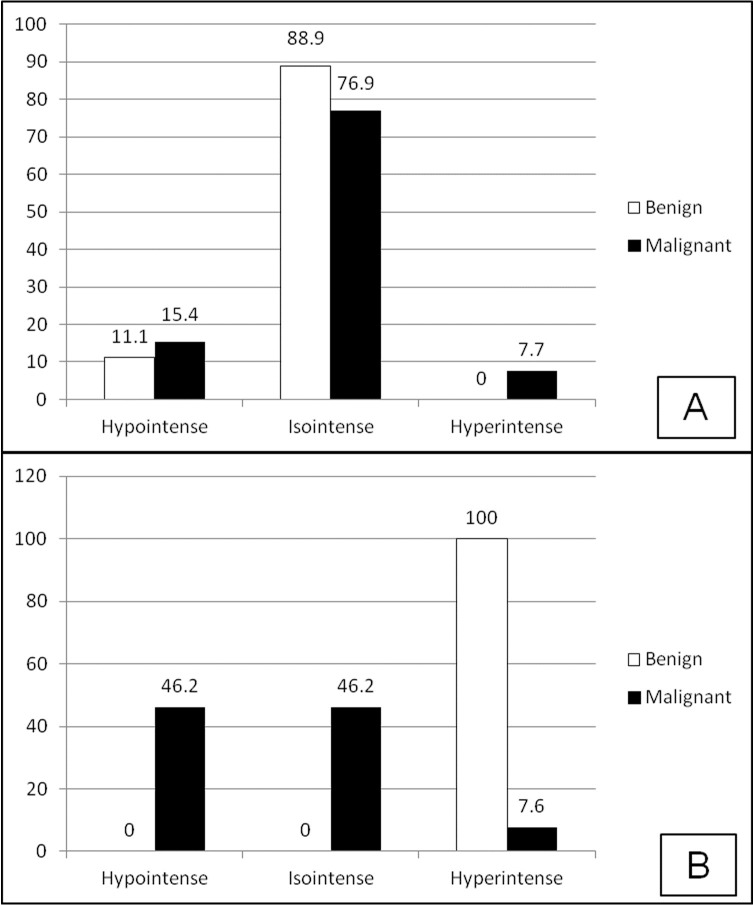
Tumor signals in magnetic resonance imaging (MRI) T1 (A) and T2 (B) weighted images.

RI and PI were measured by the ultrasonography system. Perfusion pattern was classified into the central, peripheral, and combined, and scaled into four scores as follows,^9^ (Figure 2):

- 0: no perfusion or signal flow
- +: blood vessels at gland hilar
- ++: sparse blood vessels
- +++: big or intensive blood vessels

After imaging process all patients underwent surgery and all tumors were removed and histopathological diagnoses were made. The data including tumor site, lymphadenopathy, and tumor invasion to adjacent structures were registered by the surgeon. The removed tumors were sent to the Department of Pathology for histopathological evaluations, after which the final diagnoses were registered. Tumors were categorized as either benign or malignant.

MRI, color doppler ultrasonography, and surgical data were compared. Data were analyzed using SPSS v.18.0. Chi-square statistics, Fisher’s exact test, and independent t-test were employed to analyze qualitative and quantitative data. P value ≤ 0.05 were considered as statistically significant.

## Results

The mean age of patients was 46.59±13.97 years (46.78±11.5 for females and 46.25±18.41 for males; minimum 23, maximum 67). Of 22 patients, 8 were male (36.4%) and 14 were female (63.6%). The most common diagnoses were pleomorphic adenoma (36.4%), metastases (36.4%), and mucoepidermoid carcinoma (9%). Nine tumors (40.9%) were benign and 13 (59.1%) were malignant. The prevalence of malignancy among males and females was 53.9% and 46.1%, respectively. Malignancy was significantly more prevalent among older patients (P<0.01). Common locations of tumors were parotid (86.4%), palate (9.1%), and submandibular area (4.5%).

The overall accuracy of MRI and ultrasonography in detection of tumor location was 100% and 95% (one false report of a parotid tumor), respectively. Seventeen tumors (85%) were hypoechoic and 3 (15%) had complex echo (2 metastatic cases of squamous cell carcinoma—SCC, and 1 malignant oncocytoma). No significant relation between the echo pattern and malignancy was present (P > 0.05). The majority of benign and malignant tumors were isointense in T1 images (Figure 2), while all benign tumors were hyperintense in T2 images. However, hypointense and isointense were similarly observed among malignant tumors in T2 images (Figure 2).

All malignant and benign tumors were heterogenous.

All benign tumors had well-defined borders in both MRI and ultrasonography. However, 15.4%, 61.5%, and 23.1% of malignant tumors were well-defined, ill-defined, and poorly defined in MRI evaluation, revealing 100% sensitivity. Meanwhile, ultrasonography revealed 72.7% of malignant tumors as ill-defined (Table 1). There was a significant difference between malignant and benign tumors regarding tumor border parameter (P < 0.001).

**Table 1:** Frequency of tumor border patterns in Ultrasonography among different tumors (20 tumors)

	Well-identified		Ill-identified	
Tumors	No.	%	No.	%
Pleomorphic adenoma	8	100	0	0
Warthin	1	100	0	0
Malignant oncocytoma	0	0	1	100
Adenoeid cyatic carcinoma	0	0	1	100
lymphoma	0	0	1	100
Metastatic tumors	2	25	6	75

MRI images showed no benign tumor invaded adjacent structures, while 92.3% of malignant tumors had local invasions and only one mucoepidermoid carcinoma had no local invasion. A significant relationship between malignancy and existence of local invasion was discovered (P < 0.001). Sensitivity, specificity, and positive predictive value (PPV) of local invasion for the prediction of malignancy was 92.3%, 100%, and 100%, respectively. Moreover, the accuracy of MRI in the detection of local invasion was 100%.

Lymphadenopathy was reported in 5 tumors by MRI and ultrasonography, including 3 malignant tumors (2 metastatic SCCs and 1 lymphoma) and 2 benign tumors (both pleomorphic adenomas). Of these five tumors excluding the lymphoma, the remaining 4 tumors had well-defined borders.

Regarding tumor perfusion scale, a big proportion of malignant tumors (36.3%) had ++ score, while majority of benign tumors (33.3%) were + or without signal flow (Table 2). Perfusion pattern in 50% of benign and malignant tumors was peripheral and mixed, respectively (Table 3).

**Table 2:** Frequency of perfusion scale among different tumors (20 tumors)

	No perfusion		+		++		+++	
Tumors	No.	%	No.	%	No.	%	No.	%
Pleomorphic adenoma	3	37.5	3	37.5	2	25	0	0
Warthin	0	0	0	0	0	0	1	100
Malignant oncocytoma	0	0	0	0	0	0	1	100
Adenoeid cystic carcinoma	0	0	0	0	1	100	0	0
lymphoma	0	0	0	0	0	0	1	100
Metastatic tumors	3	37.5	1	12.5	3	37.5	1	12.5

**Table 3:** Frequency of perfusion patterns among different tumors (14 tumors*)

	Central		Peripheral		Mixed (central and peripheral)	
Tumors	No.	%	No.	%	No.	%
Pleomorphic adenoma	1	20	3	60	1	20
Warthin	0	0	0	0	1	100
Malignant oncocytoma	0	0	0	0	1	100
Adenoeid cystic carcinoma	0	0	0	0	1	100
lymphoma	0	0	1	100	0	0
Metastatic tumors	1	20	2	40	2	40
^*^Six of the 20 tumors evaluated by color doppler ultrasonography were without vessels.						

Mean RI values in benign and malignant tumors were 0.77±0.1887 and 0.78±0.1167 and mean PI values were 1.71±0.9311 and 1.70±0.7815, respectively. No significant relationship was present between malignant and benign tumors regarding RI and PI (P > 0.05).

## Discussion

Various imaging techniques have been used for the evaluation of salivary gland tumors including computed tomography (CT) scan, MRI, and ultrasonography. Recently, color doppler ultrasonography was introduced as a new technique for the evaluation of vascular system in such tumors. Therefore, we conducted the present study to determine and compare MRI and color doppler ultrasonography parameters with histopathological and surgical findings as the gold standard.

To evaluate the findings, tumors were divided into benign and malignant groups. According to the previous studies and radiology textbooks,^1,7^ benign salivary gland tumors are more common and account for two thirds of all salivary gland tumors, while in our study malignant tumors were more frequent (59.1%) which may be due to the nature of patients referred to Ghaem and Omid hospitals, which tended to be more serious and complex cases.

The majority of patients in this study were female (63.6%) and the most common tumor was pleomorphic adenoma that is similar to radiology textbooks.^1,7,10^ The most common sites of tumors were parotid (86.4%), palate (9.1%), and submandibular (4.5%) areas, which were similar to the results of previous studies.^1,10^

The mean age of patients in this study was 46.59±13.97 years. Mean age of male patients with benign tumors was lower than those with malignant tumors, similar to previous reports.^1,10^

de Ru et al^11^ showed that both MRI and ultrasonography are accurate imaging techniques for localizing salivary gland tumors with respective accuracies of 100% and 90%, which is in accordance with our results. According to Brennan et al,^12^ in most patients with parotid tumors except when there is extension to deep lope or malignancy is probable, sonography is sufficient for imaging benign parotid tumors before surgery. However, ultrasonography was not very accurate in localizing deep tumors and reported them as superficial. Goto et al^13^ also reported similar results.

In the present study, the ramus of the mandible was used as an index for localizing tumors, which revealed 100% accuracy. Divi et al^14^ used retromandibular vein as the index with 95% accuracy.^14^ According to de Ru et al,^11^ MRI and palpation are more accurate in localizing tumors.^11^

Our results showed that the majority of tumors had hypoechoic patterns in sonography which was similar to previous studies.^9,15^ Moreover, 3 tumors had complex echo which also received +++ score by color doppler ultrasonography scale. White et al^7^ state that complex echo pattern is a common finding in hemangioma due to its vascular nature. Although Ishii et al^16^ claim that it is possible to differentiate tumors based on their echo pattern, our study could not replicate such a finding.^16^

All benign tumors in our study had high signals in T2 images, while only 7.7% of malignant tumors showed high signals. Also the sensitivity of hyperintense pattern in T2 images for the detection of benign tumors was 100%. According to Som et al,^1^ most of benign tumors have high signals in T2 images as a result of being well-differentiated, while malignant tumors have lower signals due to their poor-differentiation. Malignant tumors in our study had low to moderate signals in T2 images, which is similar to other studies.^17,18^ There was one lymphoma case in our study which had low signals in both T1 and T2 images and was associated with lymphadenopathy and brain metastasis. Tauber et al^19^ also reported two cases of lymphoma with low and moderate signals in T1 and T2 images.

Christe et al^18^ showed that infiltration of subcutaneous tissue is a specific sign predictive of malignancy. Other studies also reported invasion to adjacent structures as the accepted parameter for prediction of malignancy which was also confirmed by our results.^20,21^

All benign tumors in our study revealed well-defined borders, which is in line with previous reports.^1,7,10^ Ill-defined margins of a parotid tumor are highly suggestive of malignancy.^11,18^ Okahara et al^22^ suggested tumor border as the most helpful parameter for the differentiation of benign and malignant tumors in MRI. We also found a significant relationship between well-defined tumor borders and benign nature of tumors in ultrasonography.

Bradley et al^23^ reported a 3 folded more possibility of malignancy in tumors with RI > 0.8 and PI > 1.8.^23^ We did not find any significant difference regarding RI and PI between malignant and benign tumors. Schick et al^24^ concluded that although color doppler ultrasonography could not differentiate benign and malignant tumors from each other, a high systolic peak and higher perfusion rate would increase the possibility of malignancy even if the tumor was reported in conventional ultrasonography as benign.

Sladana et al^9^ reported a significant difference between the perfusion pattern of pleomorphic adenoma and other tumors (P=0.01) showing that the majority of pleomorphic adenomas (75%) had peripheral perfusion. Also 53.57% of such tumors had + perfusion score and 35.7% had ++ score. Besides, 90% of malignant tumors had central perfusion and 70% had +++ score. They similarly could not find a relation between PI and RI values and malignancy.^9^ In the present study, 37.5% of pleomorphic adenomas had + perfusion score and 25% had ++. Also, perfusion pattern for 60% of pleomorphic adenomas and 36% of malignant tumors was central.

It was not possible to represent the association of tumors and facial nerve using MRI which was due to the 0.5 Tesla power MRI system used in this study.

## Conclusions

MRI and ultrasonography have high accuracy rates in localizing salivary gland tumors. It seems ultrasonography would be an appropriate imaging technique in case of localizing intra and extra glandular tumors and could not accurately display invasion to deeper adjacent anatomic structures. Also in the absence of intraoral probe, it would be impossible to display tumors in the palate. Moreover, ultrasonography cannot show the associations between tumor and adjacent structures, and thus, it would be better to use MRI whenever tumors are big or judged to have higher possibility for malignancy. The presence of invasion to adjacent structures was seen to be an acceptable parameter in the prediction of malignancy in MRI. Also a significant relationship between well-defined tumor border and benign nature of tumors was present in ultrasonographic evaluations.

## Acknowledgement

This study was made possible by the generous support rendered by the Vice Chancellor for Research of Mashhad University of Medical Sciences, in the form of grant No. 86210, for which the authors are very grateful.
